# Regulation of Respiratory Pathways in Campylobacterota: A Review

**DOI:** 10.3389/fmicb.2019.01719

**Published:** 2019-07-30

**Authors:** Anne-Xander van der Stel, Marc M. S. M. Wösten

**Affiliations:** Department of Infectious Diseases and Immunology, Utrecht University, Utrecht, Netherlands

**Keywords:** *Campylobacter jejuni*, *Helicobacter pylori*, *Arcobacter butzleri*, *Sulfurimonas denitrificans*, *Sulfurospirillum multivorans*, *Wolinella succinogenes*, electron donor/acceptor, electron transport chain

## Abstract

The Campylobacterota, previously known as Epsilonproteobacteria, are a large group of Gram-negative mainly, spiral-shaped motile bacteria. Some members like the *Sulfurospirillum* spp. are free-living, while others such as *Helicobacter* spp. can only persist in strict association with a host organism as commensal or as pathogen. Species of this phylum colonize diverse habitats ranging from deep-sea thermal vents to the human stomach wall. Despite their divergent environments, they share common energy conservation mechanisms. The Campylobacterota have a large and remarkable repertoire of electron transport chain enzymes, given their small genomes. Although members of recognized families of transcriptional regulators are found in these genomes, sofar no orthologs known to be important for energy or redox metabolism such as ArcA, FNR or NarP are encoded in the genomes of the Campylobacterota. In this review, we discuss the strategies that members of Campylobacterota utilize to conserve energy and the corresponding regulatory mechanisms that regulate the branched electron transport chains in these bacteria.

## Introduction

The Epsilonproteobacteria no longer belong to the proteobacteria, therefore their phylum name has recently been changed in Campylobacterota (Waite et al., 2017, 2018; Parks et al., 2018). Campylobacterota can be found in diverse ecological habitats, such as deep-sea hydrothermal vents, oil fields, and the digestive tracts of animals (Eppinger et al., 2004; Nakagawa and Takaki, 2009; Waite et al., 2017; Parks et al., 2018). The deep-sea vents are reservoirs of the free-living nonpathogenic Epilonbacteraeota bacteria, where they utilize compounds in vent fluids as energy sources and compounds in seawater as electron acceptors and are able to fix inorganic carbon into organic compounds (Nakagawa et al., 2005; Nakagawa and Takaki, 2009). In some microbial communities, Campylobacterota account for 90% of total biomass. For example, *Sulfurospirillum* spp. are often the dominant species found in oil contaminated groundwater or oil fields (Nakagawa et al., 2005; Grigoryan et al., 2008; Hubert et al., 2012). Campylobacterota are also recognized as important human pathogens: half of the human population is colonized with the ulcer-causing stomach bacterium *Helicobacter pylori*, whereas *Campylobacter jejuni* is one of the most prevalent bacterial food-borne pathogens (Eusebi et al., 2014; Deng et al., 2016; Ramees et al., 2017). At first glance, no obvious relationship exists between deep-sea thermal vents and the gastrointestinal tract of animals. However, Campylobacterota, such as *Sulfurovum* spp. and *Nitratiruptor* spp., are already adapted to colonize host animals. They colonize deep-sea animals like shrimps and polychaetes as commensal or symbiont (Nakagawa et al., 2005). Most Campylobacterota are highly motile due to their polar flagella. They can also use the flagella as micro-mixer to generate a constant flow of nutrients into their microenvironment (Beeby, 2015). Chemo- and energy taxis allow them to find more beneficial growth conditions. There are clear links between metabolism and motility as most chemoattractants are used as carbon source or electron donors/acceptors (Hartley-Tassell et al., 2010; Cerda et al., 2011; Rahman et al., 2014). Energy conservation enzymes like hydrogenase and the periplasmic nitrate reductase enzymes are also conserved in these organisms and are crucial for survival in their environment (Olson and Maier, 2002; Simon and Klotz, 2013; Han and Perner, 2014; Vetriani et al., 2014). Most Campylobacterota have a large and remarkable repertoire of electron transport chain enzymes, considering their small genomes. However, the mechanisms and rationale of its regulation are poorly understood (Cook et al., 2014). Here, we review what is known about the regulation of energy metabolism of six representatives Campylobacterota.

## Energy Conservation

**Figure 1:**
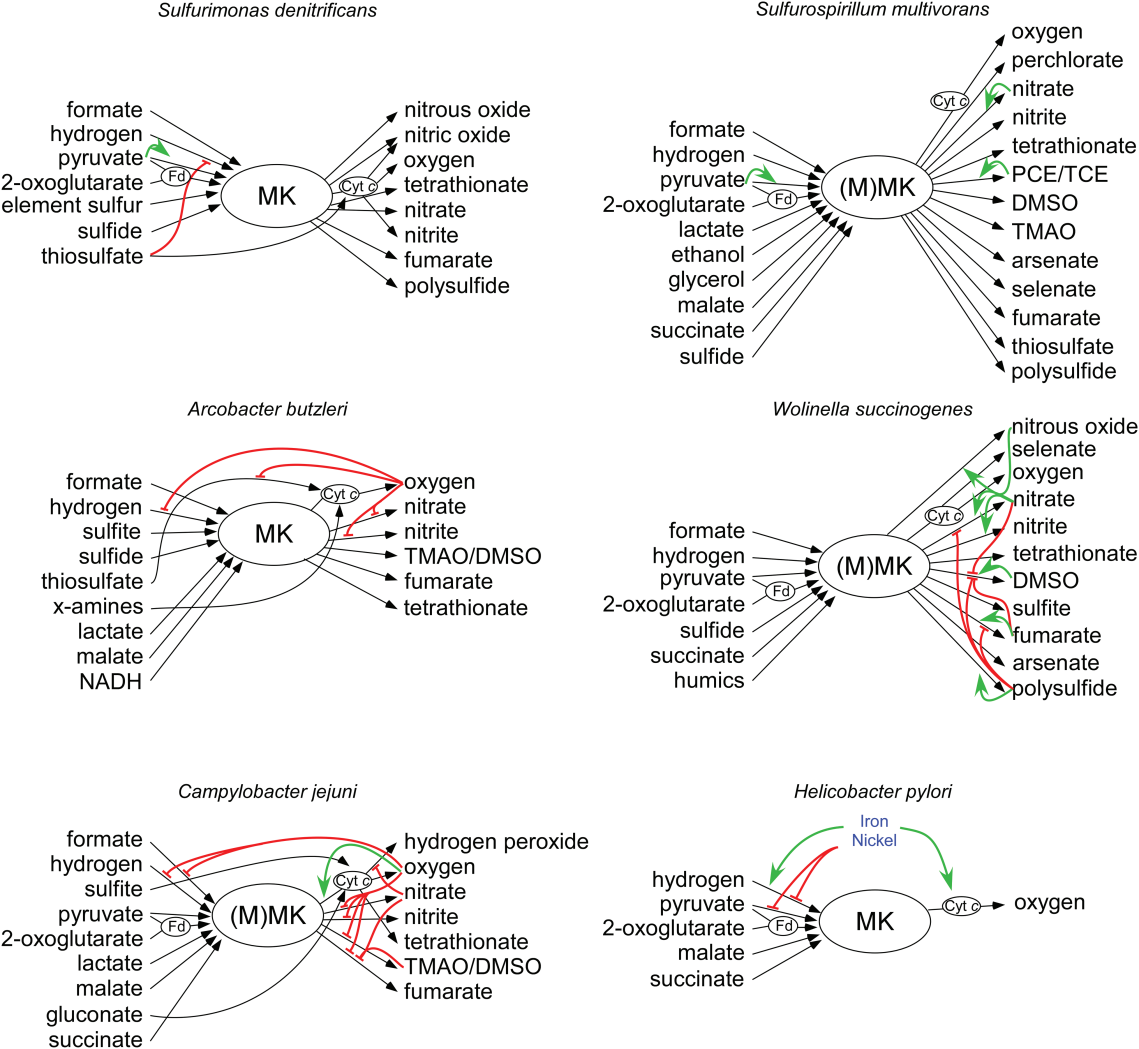
Overview of electron donors and acceptors that can be used by the six described Campylobacterota. Electron donors are depicted at the left side, and electron acceptors are located at the right side. Black arrows depict electron transferring redox enzymes. Electron shuttling compounds and proteins Fd and cyt c are depicted with an oval. Green and red arrows show the transcriptional activation and repression, respectively. Details are explained and referenced in the main text. MK, menaquinone; MMK, methyl-substituted menaquinone; Fd, ferrodoxin or flavodoxin; cyt c, cytochrome c.

To conserve energy, bacteria utilize redox reactions to build up an ion gradient (proton or sodium motive force). The proton motive force generated by Campylobacterota has several important functions (Cook et al., 2014). It is used to rotate the flagella, to generate ATP and is necessary as driving force for transport of chemicals over the membrane. The main constituent of the proton motive force is the membrane potential (Δψ), which is produced during respiration by specialized redox enzymes, together forming the electron transport chain (ETC) (Mitchell, 1961; Simon et al., 2008). Some membrane proteins of the ETC function as proton pumps and can directly pump protons from the cytoplasmic side to the periplasmic side of the membrane. Another mechanism to generate a membrane potential is *via* a redox loop. During this process, electron transfer from the electron donor to the terminal electron acceptor complex is facilitated by membrane-associated molecules called quinones and two (or more) redox proteins. Quinones freely diffuse through the membrane, and when fully reduced, they carry two electrons and two protons. Reducing quinones on the cytoplasmic side and oxidizing quinols on the periplasmic side enable electron (and proton) translocation over the membrane (Simon et al., 2008). Different quinones, with different redox properties are produced dependent on the electron acceptor that is used to efficiently facilitate electron transport from the electron donor to the terminal electron acceptor. The Gammaproteobacterium *Escherichia coli* possesses three different quinones; under aerobic growth, it uses ubiquinone, which is replaced under anaerobic growth by menaquinone (MK) or demethylmenaquinone when fumarate, or nitrate, respectively, is present as electron acceptor (Unden and Bongaerts, 1997). Campylobacterota lack ubiquinone and demethylmenaquinone, but they all produce MK with a redox potential of *E*′° = −75 mV (Carlone and Anet, 1983; Guccione et al., 2010). Some Campylobacterota, like *Wolinella succinogenes* and *Campylobacter jejuni* also produce a methyl-substituted menaquinone (MMK), which has a lower redox potential of *E*′° = −124 mV (Carlone and Anet, 1983; Juhnke et al., 2009; Hein et al., 2017a,b). MMK is synthetized by MqnK, a methyltransferase enzyme that uses MK as a substrate. In *W. succinogenes* and *C. jejuni*, MMK serves as a redox partner for a periplasmic fumarate reductase complex named Mfr (Juhnke et al., 2009). MMK probably also allows respiration with molecules with a low redox potential such as polysulfide or sulfite Figure 1; (Hein, et al., 2017a). So far no functional distinction has been made between these different quinones, although some reductases seem to have a higher catalytic activity with MMK than with MK (Dietrich and Klimmek, 2002; Juhnke et al., 2009). Campylobacterota can utilize various molecules as electron acceptors by using different specialized enzyme complexes (Figure 1). Campylobacterota are microaerophilic organisms that cannot survive atmospheric oxygen tensions due to oxygen sensitive iron-sulfur clusters of the atypical enzymes pyruvate:flavodoxin oxidoreductase (POR) and 2-oxoglutarate:acceptor oxidoreductase (OOR) (which oxidize pyruvate and 2-oxoglutarate, respectively) (Kendall et al., 2014). However, oxygen is a preferred electron acceptor for many Campylobacterota (Kelly, 2001; Sellars et al., 2002). Enzymes like the bacterial cyt *bc*_1_-complex use a process called Q-cycling to translocate protons (and electrons) over the membrane by simultaneously reducing quinones on the cytoplasmic side and oxidizing quinols on the periplasmic side of the membrane (Brandt and Trumpower, 1994). Campylobacterota encode an ancient form of the *bc*_1_ complex, a Riekse/cyt *bc* complex, which has been shown to also play a role in anaerobic respiration (Baymann et al., 2004; Hein, et al., 2017a; Garg et al., 2018). When oxygen is used as electron acceptor, electrons are transferred from the MK pool through the proton translocating Riekse/cyt *bc* complex (encoded by the *qcrABC* genes, previously *petABC*), where they are used *via* a periplasmic cyt *c* protein to reduce the cyt *cbb*_3_ complex, a proton pumping oxidase (Tsukita et al., 1999; Jackson et al., 2007; Tanigawa et al., 2010). Due to these two proton translocating enzyme complexes, oxygen respiration yields a membrane potential, which leads to a higher growth rate than with other electron acceptors (van der Stel et al., 2017). Apart from respiration with oxygen, respiration with other compounds like fumarate, nitrate, nitrite, nitrous oxide, TMAO/DMSO or sulfur compounds (e.g., elemental sulfur, polysulfide, sulfite, tetrathionate, and thiosulfate) is possible, if the specific reductase is present in the bacterium. Most of these reductases do not contribute to the generation of the membrane potential but only function as electron sinks (Biel et al., 2002; Pittman and Kelly, 2005; Guccione et al., 2010; Hermann et al., 2015). In this case, the generation of the membrane potential is dependent on the donor complex (Simon et al., 2008). Various carbon sources can also act as electron donors. Therefore, carbon and energy metabolism, as well as its regulation is inherently linked. Electrons liberated from pyruvate and 2-oxoglutarate oxidation by POR and OOR, respectively, are shuttled to the NADH:ubiquinone oxidoreductase (Nuo) complex (Biel et al., 1996; Hughes et al., 1998; Parkhill et al., 2000; St Maurice et al., 2007). The Nuo complex found in Campylobacterota (εNuo) is a proton pumping enzyme complex that reduces MK (Weerakoon and Olson, 2008). The *ε-nuoA-N* gene cluster is homologous to those of other organisms, except for the *nuoEF* genes, which are replaced with two other genes (*nuoXY*) and likely facilitate the redox coupling with the proteins flavodoxin or ferredoxin instead of the canonical NADH (Weerakoon and Olson, 2008). Oxidation of other carbon sources, such as succinate, malate, and lactate, is also directly coupled to MK reduction (Hoffman and Goodman, 1982). Some molecules do not serve as carbon source, but are utilized as electron donor only. Formate and molecular hydrogen are excellent electron donors, because of their low mid-point redox potential. Most Campylobacterota encode at least one hydrogenase and one formate dehydrogenase (Figure 1; Laanbroek et al., 1978; Hoffman and Goodman, 1982; Olson and Maier, 2002; Takai et al., 2005; Marreiros et al., 2016). The enzymes that oxidize hydrogen or formate have an active site in the periplasm and a membrane-spanning subdomain that interacts with menaquinone near the cytoplasm. This makes these enzymes electrogenic *via* the redox-loop mechanism (Biel et al., 2002; Jormakka et al., 2002). Other molecules that are exploited as electron donor include reduced sulfur compounds like sulfite, sulfide, and tetrathionate, as well as gluconate (Campbell et al., 2006). The versatility of electron donors and acceptors that can be used by individual organisms refers to the presence of these molecules in the environments of these bacteria and suggests the need for regulatory mechanisms to optimize energy conservation.

## Regulation of Respiratory Metabolism

Bacteria have different strategies to adapt to the presence of different metabolic substrates. Often an enzyme is only expressed in the presence of its substrate, or conversely when all other possible substrates are exhausted (Unden and Bongaerts, 1997; Golby et al., 1999). This requires specific sensor and regulator proteins for each substrate. The respiration enzymes in many bacteria are hierarchically regulated by a large number of transcription factors (Unden and Bongaerts, 1997; Arai, 2011; Hartig and Jahn, 2012). However, for Campylobacterota multiple studies have shown that co-respiration takes place, indicating that in these bacteria, the regulation of the electron transport chain is subjected to more global cues from the environment (Lorenzen et al., 1993; Weingarten et al., 2008; Dahle et al., 2013; Goris et al., 2015). In Firmicutes, like *Staphylococcus aureus* and *Bacillus subtilis*, the nitrate dehydrogenase complexes are induced upon low oxygen tension by the ResDE two-component system and the transcriptional regulator FNR, independent of the presence of nitrate (Fuchs et al., 2007; Hartig and Jahn, 2012). Rex responding to intracellular redox potential is a central regulator of anaerobic metabolism in *S. aureus*, and orthologues have been identified in the phyla Actinobacteria *Thermotogales*, *Actinobacteria*, *Chloroflexi*, *Deinococcus-Thermus*, and *Proteobacteria* (Ravcheev et al., 2012). In model organisms like *E. coli* as well in many other Gammaproteobacteria, multiple global and local regulatory proteins such as ArcA, FNR, DcuRS, and NarPL have been identified that help these bacteria to adapt to the presence of different electron acceptors (Ravcheev et al., 2007). For example, low oxygen tension and the presence of the alternative electron acceptor nitrate are necessary to induce the nitrate respiratory machinery (Unden and Bongaerts, 1997; Goh et al., 2005; Arai, 2011). These regulatory systems are widely distributed and conserved in the Proteobacteria, but not in the Campylobacterota (formerly Epsilonproteobacteria) (Leyn et al., 2016). Genome and mutagenesis analyses indicate that the genes encoding these and other well-studied regulators are lacking in the genomes of Campylobacterota (Tomb et al., 1997; Parkhill et al., 2000; Baar et al., 2003; Miller et al., 2007; Sievert et al., 2008; Goris et al., 2014; De la Cruz et al., 2017; van der Stel et al., 2018). Campylobacterota have relatively small genomes compared to other bacteria and consequently a scaled down number of regulatory proteins (Galperin, 2006). This makes it difficult to predict the behavior of these organisms in different growth conditions and leaves metabolic regulation an enigma. Here, we review the current state of knowledge about the regulation of energy metabolism of six Campylobacterota species that represent the Campylobacterota from a wide range of different habitats.

## Respiratory Pathways of Free-Living Marine Campylobacterota

Free-living marine Campylobacterota such as *Sulfurospirillum* and *Sulfurimonas* spp. are generally associated with sulfide rich environments, where they play a key role in the cycling of carbon, nitrogen, and sulfur (Table 1; Campbell et al., 2006). Some of these bacteria as well as the deep-sea symbionts *Sulfurovum* spp. and *Nitratiruptor* spp. contain all the genes necessary for a reductive TCA-cycle (rTCA), which is used to incorporate CO_2_ as carbon source (Hugler et al., 2005; Sievert et al., 2008; Glaubitz et al., 2009; Goris et al., 2014). The switching of the direction of the TCA cycle in Campylobacterota has not been studied, but is likely to occur since all enzymes can run in both the oxidative and the reductive direction. Both enzymes POR and OOR catalyze reversible decarboxylation reactions, while the enzyme FrdABC, which is linked to the menaquinone pool, can function as both succinate dehydrogenase and fumarate reductase (Lancaster and Simon, 2002; Weingarten et al., 2009). The enzyme ATP citrate lyase (AclAB), which is present in most known marine Campylobacterota, catalyzes the formation of acetyl-CoA from citrate and seems to be an indicator for autotrophic CO_2_ fixation for Campylobacterota (Nakagawa and Takaki, 2009). The presence of ATP citrate lyase may reflect the absence of other, more complex, carbon sources in marine environments, like sugars, amino acids, and oxoacids that are used by host-associated Campylobacterota. Despite the presence of a relative high number of regulators and elaborate electron transport chains (Table 1, Figure 1), there is little knowledge of the regulation of metabolism in the free-living Campylobacterota (Figure 1).

**Table 1:** The number of transcription factors and the occurrence of the *aclA* gene in different Campylobacterota and for comparison also in other bacteria.

Species	Habitat	Genes	*aclA*	TF	TF/per 1,000 genes
*Sulfurimonas denitrificans*	Free living	2,192	Yes	53	24.2
*Sulfurimonas autotrophica*	Free living	2,198	Yes	50	22.7
*Sulfurospirillum multivorans*	Free living	3,268	Yes	121	37.0
*Sulfurospirillum barnesii*	Free living	2,527	No	61	24.1
*Arcobacter nitrofigilis*	Free living	3,155	No	143	45.3
*Arcobacter butzleri*	Free living/host-associated	2,327	No	102	43.8
*Nitratiruptor sp.*	Free living/host-associated	1,969	Yes	43	21.8
*Sulfurovum sp.*	Free living/host-associated	2,551	Yes	73	28.6
*Nautilia profundicola*	Free living/host-associated	1,730	No	35	20.2
*Wolinella succinogenes*	Host-associated	2,092	No	70	33.5
*Campylobacter jejuni*	Host-associated	1,628	No	28	16.7
*Campylobacter coli*	Host-associated	1,602	No	25	15.6
*Helicobacter hepaticus*	Host-associated	1,916	No	32	16.7
*Helicobacter pylori*	Strictly host-associated	1,610	No	12	7.5
*Bacillus subtilis*	Free living	4,381	No	280	63.9
*Caulobacter crescentus*	Free living	3,969	No	230	57.9
*Myxococcus xanthus*	Free living	7,262	No	302	41.6
*Streptomyces coelicolor*	Free living	7,851	No	804	102.4
*Escherichia coli*	Free living/host-associated	4,497	No	293	65.2
*Enterococcus faecalis*	Free living/host-associated	2,965	no	210	73.0
*Clostridium perfringens*	Free living/host-associated	2,876	No	155	53.9
*Bacteroides fragilis*	Strictly host-associated	4,577	No	198	43.3
*Chlamydia trachomatis*	Intracellular	931	No	12	14.0
*Rickettsia rickettsii*	Intracellular	1,308	No	22	16.8

*aclA, ATP citrate lyase gene; TF, predicted number of transcription factor proteins. Reference strains used: Sulfurimonas denitrificans DSM 1251; Sulfurimonas autotrophica DSM 16294; Sulfurospirillum multivorans DSM12446; Sulfurospirillum barnesii SES-3: Arcobacter nitrofigilis DSM 7299; Arcobacter butzleri RM4018; Wolinella succinogenes DSM 1740; Campylobacter jejuni NCTC 11168; Campylobacter coli 76,339; Helicobacter hepaticus ATCC 51449; Helicobacter pylori 26,695; Bacillus subtilis 168; Caulobacter crescentus NA1000; Myxococcus xanthus DK 1622; Streptomyces coelicolor A3(2); Escherichia coli MG1655; Enterococcus faecalis D32; Clostridium perfringens ATCC 13124; Bacteroides fragilis YCH46; Chlamydia trachomatis F/11-96; Rickettsia rickettsii str. Iowa. Number of genes is taken from Biocyc (https://biocyc.org/); TF were determined using P2TF (www.p2tf.org/) (Ortet et al., 2012) or BLAST for S. multivorans*.

*Sulfurimonas denitrificans* was originally isolated from marine coastal sediments, but is known to be present in diverse marine environments (Timmer-TenHoor, 1975; Han and Perner, 2015). Due to the energy taxis, which sense the metabolic status of the cell, they are mainly found in the redox gradient zone where electron donors and acceptors are present (Grote et al., 2012). *S. denitrificans* can grow with a variety of electron donors and acceptors. Due to the presence of sulfide:quinone reductase (SQR, EC 1.8.5.4) and *sox* genes, many kinds of reduced sulfur compounds, such as sulfide, elemental sulfur, and thiosulfate can serve as electron donor, which are released from deep-sea hydrothermal vents (Nakagawa and Takaki, 2009). The electron donors pyruvate and 2-oxoglutarate can be oxidized due to the present of the *S. denitrificans* POR and OOR complexes which shuttle the electrons to the Nuo complex (Hugler et al., 2005; St Maurice et al., 2007). Apart from metabolites used in carbon metabolism, *S. denitrificans* also encodes the respiratory enzymes, FdhABC and HydABC, which enable the use of formate and hydrogen as electron donors, respectively (Sievert et al., 2008; Han and Perner, 2014, 2015). The hydrogenase activity is repressed in the presence of thiosulfate, whose oxidation is directly coupled to cyt *c* (Figure 1), indicating a preference for the latter respiratory pathway over the former. Indeed, thiosulfate leads to a faster initial growhtrate, although hydrogen as electron donor gives a higher final cell density (Han and Perner, 2014). *Sulfurimonas denitrificans* can use oxygen, fumarate, nitrate, nitrite, and polysulfide as electron acceptors and has the ability to reduce nitrite further to NO, N_2_O, and finally N_2_ (Figure 1; Sievert et al., 2008; Goris et al., 2014). The nitrite and nitric oxide reductases (NorBC and NirS, respectively) receive their electrons from cognate cyt *c* proteins *via* the Riekse/cyt *bc* complex, indicating that these reductases could contribute to the generation of the membrane potential (Figure 1; Sievert et al., 2008). *S. denitrificans* possesses only 53 putative transcription factors which might explain why the expression of the donor and acceptor complexes in *S. denitrificans* thus far seems to be mostly constitutive under aerobic and anaerobic conditions, suggesting that co-respiration of multiple chemical energy sources takes place simultaneously (Table 1; Sievert et al., 2008; Dahle et al., 2013). This constitutively expressed regulation might indicate a relatively stable supply of these compounds in their natural environment.

*Sulfurospirillum multivorans* is known that it inhabits a wide range of microbial habitats where sulfide containing water or sediments is in contact with oxic water (Scholz-Muramatsu et al., 1995; Nakagawa and Takaki, 2009). In addition to a MK, BlastP results suggest that *S. multivorans* may possess a MMK like in *W. succinogenes* and *C. jejuni* (Hein, et al., 2017a). It has been shown that under microaerobic conditions, *S. multivorans* grows by respiratory oxidation of succinate, fumarate, malate, lactate, pyruvate, and 2-oxoglutarate (Nakagawa and Takaki, 2009; Goris et al., 2014). Other electron donors that are used are hydrogen, formate, ethanol, and glycerol (Scholz-Muramatsu et al., 1995). When hydrogen gas is used as electron donor acetate, but not carbon dioxide, is required as the carbon source (Nakagawa and Takaki, 2009). Like *S. denitrificans, S. multivorans* is also able to reduce and oxidize sulfur compounds and reduce nitrate to nitrite or ammonium compounds (Scholz-Muramatsu et al., 1995; Luijten et al., 2003). But in contrast to *S. denitrificans*, it can also reduce various toxic compounds (arsenate and selenate), sulfur compouds (thiosulfate and tetrathionate), S/N-oxides (TMAO and DMSO), and organohalides [tetrachloroethene perchloroethylene (PCE) and trichloroethene (TCE)], which are all coupled to MK (Nakagawa and Takaki, 2009; Goris et al., 2014). Some of these genes are likely aquired horizontally and support the bacteria survival in polluted waste water, from where it was first isolated. The multitude of respiratory enzymes makes S. multivorans extremely versatile. Along with the extensive electron transport chain comes extensive regulation as *S. multivorans* possesses 2.5 times more transcription factors than *S. denitrificans* (Table 1). The nitrate reductase complex Nap of *S. multivorans* seems to be regulated by different growth conditions, but not by its substrate nitrate (Figure 1; Goris et al., 2015). The main hydrogen oxidizing enzyme (the membrane bound hydrogenase HydABC) and the main formate dehydrogenase (Fdh2) are constitutively expressed, indicating an important role for these enzymes (Goris et al., 2015; Kruse et al., 2017). A hydrogen producing enzyme (Hyf) is repressed in the presence of an electron acceptor, indicating a possible role in redox-balancing under fermentative conditions (Kruse et al., 2017). The expression of the tetrachloroethylene (PCE) dehalogenase is induced by its substrate PCE and seems to be dependent on a two-component regulatory system (Turkowsky et al., 2018). It is remarkable that in the absence of PCE, it takes more than 100 generations to downregulate the PCE respiration. It was recently hypothesized that acetylation of a response regulator might be the cause of this unique long-term down-regulation of gene expression (John et al., 2009; Turkowsky et al., 2018). This introduces a new form of long-term, memory-like, regulation.

## Respiratory Pathways in Host-Associated Campylobacterota

More research has been performed on the regulation of the respiratory pathways in the known host-associated Campylobacterota. The non-marine host-associated Campylobacterota have lost the ability to fix CO_2_ as they miss the *acl*AB genes necessary to allow the reductive TCA cycle (Waite et al., 2017). Therefore, these organisms rely on organic carbon sources. The host-restricted Campylobacterota possess less regulatory proteins and have lost some respiratory pathways (Table 1). Here, we discuss the current knowledge of the metabolic regulation in the species *Arcobacter butzleri*, *Wolinella succinogenes*, *Campylobacter jejuni*, and *Helicobacter pylori*.

*Arcobacter butzleri* is present in the environment as well as in the gastrointestinal tracts of animals and humans (Ho et al., 2006). *A. butzleri* possesses a similar electron transport chain as the free-living marine Campylobacterota and can utilize a large number of electron donors and acceptors (Figure 1). However, in contrast to other Campylobacterota, *A. butzleri* is well equipped to survive under higher atmospheric oxygen levels. *A. butzleri* contains an oxygen-stable pyruvate kinase instead of the oxygen-labile POR enzyme and also contains the canonical *nuoEF* genes instead of the Campylobacterota specific *nuoXY* genes (Miller et al., 2007). Based on genome analysis and for some compounds experimental proof, they can use H_2_, formate, sulfite, sulfide, thiosulfate, x-amines, lactate, malate, and NADH as electron donors and oxygen, nitrate, nitrite, TMAO/DMSO tetrathionate, and fumarate as electron acceptors (Miller et al., 2007; Martinez-Malaxetxebarria et al., 2012; Liu et al., 2013; Han and Perner, 2015; Kurth et al., 2017). Of the host-associated Campylobacterota, *A. butzleri* possesses the highest number (102) of regulatory genes (Table 1). Among them are seven extracytoplasmic function sigma-factors that are activated by a specific intra- or extracellular stimuli, suggesting specific adaptation capabilities needed to survive in multiple environments (Helmann, 2002; Miller et al., 2007). Four of these seven extracytoplasmic function sigma-factors (ECF) impact on electron and carbon metabolism, by affecting the expression of electron donor complexes, acetate pathway genes (*ackA* and *pta*), and electron acceptor complexes as shown by (Martinez-Malaxetxebarria et al., 2012). Adaptation toward changing redox conditions might very well be regulated by the six histidine kinases that contain PAS-domains, known to respond to oxygen and redox signals (Miller et al., 2007). It is noteworthy that this organism does not encode the post-transcriptional regulator *csrA*, nor a homologue of the FNR/CRP-family regulators like most other Campylobacterota (Miller et al., 2007). No functions are reported yet for the other regulatory proteins of *Arcobacter.*

*Wolinella succinogenes* is found colonizing the bovine rumen as a commensal (Vandamme et al., 1992; Simon et al., 2006). Probably due to the more restricted environment it occupies, a reduced number of 70 transcription regulator genes are found in the genome of *W. succinogenes* (Table 1; Baar et al., 2003). This restricted environment however contains a large variety of electron donor and acceptors as *W. succinogenes* can use formate, hydrogen, sulfide, pyruvate 2-oxoglutarate, succinate, and even humics as electron donor and NO, O_2_, nitrate, nitrite tetrathionate, DMSO, sulfite, fumarate, arsenate, polysulfide, and selenate as electron acceptor (Lorenzen et al., 1993, 1994; Biel et al., 1996; Lovley et al., 1999; Kroger et al., 2002; Kern et al., 2011; Kurth et al., 2017). Transcription of the *W. succinogenes* complexes that reduce fumarate (Frd), nitrate (Nap), polysulfide (Psr), and DMSO (Dms) are all induced when cultures are grown with their corresponding substrate as electron acceptor (Figure 1; Lorenzen et al., 1993, 1994; Kern and Simon, 2016). *W. succinogenes* is able to respond to the presence of nitrate, nitric oxide (NO) or nitrous oxide (N_2_O) in the environment in which three Nss type transcription regulator proteins NssA, NssB, and NssC are involved (Kern and Simon, 2016). NssA upregulates the nitrate-, nitrite- and nitrous oxide reductases when the bacteria are grown in the presence of nitrate. The same reductases are upregulated by NssB and NssC, which are activated in response to NO and N_2_O, respectively (Kern and Simon, 2016). A similar response may occur in the free**-**living marine Campylobacterota as they contain similar genes in their genomes (Kern and Simon, 2016). PsrR, a member of the AraC family of transcription factors regulates the polysulfide reductase complex PsrABC allowing *W. succinogenes* to use polysulfide as electron acceptor (Braatsch et al., 2002). PsrR is probably regulated by nitrate or nitrite as in the absence of polysulfide, nitrate or nitrite represses the formation of the polysulfide reductase enzyme (Braatsch et al., 2002). Besides substrate-induced upregulation of respiratory complexes, a hierarchical use of electron acceptors is present in *W. succinogenes*. DMSO respiration is repressed by the presence of both fumarate and nitrate, which also lead to a higher growth yield (Lorenzen et al., 1994). The presence of electron acceptors exerts repression of the utilization of other electron acceptors that are lower on the hierarchical ladder (Unden and Bongaerts, 1997). In the case of *W. succinogenes,* polysulfide (−270 mV) represses respiration with fumarate, nitrate, and DMSO. This is remarkable, because polysulfide yields the least amount of ATP per electron and results in a lower growth yield compared to other electron acceptors, but does result in a higher growth rate (Macy et al., 1986; Unden and Bongaerts, 1997). Such a mechanism to favor growth rate over yield might be beneficial when competing with the microbiota in the bovine rumen (Foster et al., 2017).

*The human pathogen Campylobacter jejuni* is a major cause of acute bacterial diarrhea, but in a wide variety of domestic and wild animals, it is a commensal of the gastrointestinal tract (Bronowski et al., 2014; EFSA, 2017). Besides that *C. jejuni* colonizes the gastrointestinal tract of warm-blooded animals, it is able to survive in surface water (Horrocks et al., 2009; Bronowski et al., 2014). Although *C. jejuni* possesses a small genome and in contrast to the free-living Campylobacterota has lost most of its regulatory proteins, it still has a branched electron transport chain with multiple redox-enzymes that utilize an array of molecules as electron donor and acceptor (Table 1, Figure 1). *C. jejuni* is able to use the same electron acceptors as *A. butzleri* and even hydrogen peroxide (Sellars et al., 2002; Myers and Kelly, 2005; Kelly, 2008). Formate, hydrogen, sulfite, pyruvate, 2-oxoglutarate, lactate, malate, gluconate, and succinate are used as electron donors (Myers and Kelly, 2005; Pajaniappan et al., 2008; Weerakoon and Olson, 2008; van der Stel et al., 2017). In contrast to the free-living Campylobacterota, no substrate specific reductase induction is observed in *C. jejuni* (van der Stel et al., 2017). Although *C. jejuni* is sensitive to atmospheric oxygen levels, oxygen is the preferred electron acceptor (van der Stel et al., 2017). When oxygen is unavailable, the transcription of all genes coding for alternative electron acceptor complexes such as nitrate and fumarate reductases are upregulated as well as the genes coding for the electron donor complexes, formate dehydrogenase, and hydrogenase (van der Stel et al., 2017). Also the enzyme AspA, which produces intracellular fumarate by deamination of aspartate, is upregulated in the absence of oxygen. Fumarate is subsequently used as electron acceptor by FrdABC at the expense of succinate excretion (Guccione et al., 2010; Liu et al., 2012; van der Stel et al., 2015). Although many regulators of *C. jejuni* are characterized, no oxygen responsive regulator has been identified (van der Stel et al., 2018). Under low oxygen conditions when the electron acceptors nitrate or TMAO are present, the *aspA* gene is once again repressed. This is achieved by the unique two-component system RacRS (van der Stel et al., 2015). This system of which the specific signal that activates the sensor RacS is unknown, represses the uptake and production of fumarate to prevent the use of fumarate as electron acceptor (van der Stel et al., 2015). Interestingly, a cyt *c* peroxidase (Cj0358c) is also repressed by RacR. This peroxidase does not confer resistance to hydrogen peroxide, but could play a role in respiration of hydrogen peroxide, as was discovered for other intestinal bacteria (Flint et al., 2014; Khademian and Imlay, 2017). In contrast to RacR, the LysR-type regulator Cj1000 activates the gene transcription of the uptake (*dcuA*), production (*aspA*), and respiration (*frdA/B*, *mrfA/B*) of fumarate (Dufour et al., 2013). Furthermore, Cj1000 also activates the respiration using sulfite (Dufour et al., 2013). Because the specific signals that activate the RacRS system and the Cj1000 transcription factor are unknown, it is unclear how exactly these alternative electron acceptor pathways are regulated. The post-transcriptional regulator CsrA also seems to control aspects of cellular respiration especially at the stationary phase as mutations of this gene lead to a reduced amount of respiration-related proteins MfrA, TorA, and Cj0414 (Guccione et al., 2010; Liu et al., 2014; Fields et al., 2016). The expression of formate dehydrogenase in *C. jejuni* is upregulated during colonization of chickens, but is surprisingly downregulated during colonization of human volunteers (Crofts et al., 2018). Expression of formate dehydrogenase ensures the generation of the proton motive force in the absence of oxygen, but it requires selenium as it contains a selenocysteine residue (Shaw et al., 2012; van der Stel et al., 2017). The expression of formate dehydrogenase is controlled at the posttranscriptional level by the accessory proteins FdhTU, which contribute to the acquisition or use of selenium (Shaw et al., 2012). Despite many years of research, it is still unclear what the exact regulatory mechanisms are that *C. jejuni* uses to adapt to the intestinal environment and how this is linked with its pathogenicity.

*Helicobacter pylori* is a human pathogen associated with duodenal and gastric ulcers and that solely colonizes the mucosal surfaces of the stomach (Rimbara et al., 2011). *H. pylori* is able to move to and colonize a specific zone in the gastric mucus by sensing the intracellular redox conditions in the host by energy taxis protein TlpD (Schreiber et al., 2004). In contrast to the environmental Campylobacterota, the strictly host-associated bacterium *H. pylori* co-evolved with humans for thousands of years and lost nearly all its metabolic regulators, along with many electron transport chain genes (Table 1; Schreiber et al., 2004). *H. pylori* is unable to respire with other electron acceptors than oxygen or fumarate (Hazell and Mendz, 1997; Myers and Kelly, 2004). The bacterium harbors a hydrogenase, which is indispensable for efficient pathogenicity (Nedenskov, 1994; Olson and Maier, 2002; Wang et al., 2016). Furthermore, it is able to use pyruvate, 2-oxo-glutarate, malate, and succinate as electron donors (Hughes et al., 1998; Kelly et al., 2001; Myers and Kelly, 2004; Weerakoon and Olson, 2008). Regulation of electron transport chain genes in *H. pylori* seems to be mostly regulated by the presence of metal co-factors (Haley and Gaddy, 2015). In the absence of iron, the transcriptional regulator Fur represses the transcription of HydABC (containing three iron-sulfur clusters) and cyt *c* (containing a prosthetic heme group) (Merrell et al., 2003; Carpenter et al., 2013). At the same time, the *hydABC* operon (encoding the [NiFe]-hydrogenase) is regulated by the nickel sensitive two-component system NikRS (Contreras et al., 2003). This all indicates that *H. pylori* only regulates its hydrogenase and other respiratory pathways as a result of availability of necessary co-factors iron and nickel, rather than metabolic cues (Figure 1).

## Discussion and Future Directions

Instead of a hierarchal regulation of the respiration enzymes by a large number of transcription factors as is seen in *E. coli* and other bacteria (Table 1; Unden and Bongaerts, 1997; Arai, 2011; Hartig and Jahn, 2012), Campylobacterota possess a less sophisticated regulation of their branched electron transport chains. Adaptation to a host in a number of Campylobacterota has led to the loss many genes including metabolic regulators genes as the host provides a steady predictable supply of energy substrates (Moran, 2002; Koskiniemi et al., 2012). Instead of using multiple global and local regulatory proteins, the branched electron transport chain in Campylobacterota is subjected to more global cues from the environment. This is in line with the complexity of respiratory routes that correlates with the lifestyle of the Campylobacterota (Figure 1, Table 1). The respiratory behavior of these organisms can thus make us understand more about a bacterium lifestyle and potentially why, how, and when a bacterium becomes pathogenic.

There is little conservation between the regulatory proteins of the Campylobacterota, which resembles the high mutagenic and evolutionary rate of this phylum (Porcelli et al., 2013). Nevertheless, two main strategies of electron transport chain regulation can be distinguished: first, the repression of reductases that are lower on the redox hierarchical ladder and second, the substrate dependent induction of a specific reductase. Campylobacterota seem to rely mostly on the ladder mechanism. In the absence of the preferred electron acceptor, all other reductases seem to be expressed, irrespective of the presence of their cognate substrates. As a result, co-respiration of several chemical energy sources is likely to be a common event in Campylobacterota (Lorenzen et al., 1993; Weingarten et al., 2008; Dahle et al., 2013; Goris et al., 2015); a phenomenon also observed in some other bacteria (Fuchs et al., 2007; Chen and Strous, 2013), indicating that this represents an evolutionary beneficial method to efficiently adapt to environmental electron acceptors. There are observations of species prioritizing a fast growth rate over a higher growth yield (Lele and Watve, 2014). This behavior indicates that respiratory substrates are probably only temporarily available, and fast growth burst are the best strategy to gain an advantage over competing microorganisms (Foster et al., 2017). Another remarkable similarity is the conservation of formate and hydrogen respiratory enzymes in Campylobacterota. These donors, with low redox potentials are also implicated as essential for survival under certain (anoxic) conditions.

It is clear from this report that more data are needed, especially from the free-living Campylobacterota, to get a deeper insight how these bacteria regulate their electron transport chains. Several fundamental questions are still unanswered, such as what are the exact signals and mechanism that these bacteria use to adapt to the environment. There is a clear link between chemotaxis and respiration, since many chemoattractants are metabolic substrates and bacteria often cumulate inside optimal respiratory zones, but how they are mechanistically and molecularly linked is not known. Transcriptomics, proteomics, and metabolomics data obtained by growing these bacteria and appropriate derived mutants in the presence of different electron acceptors/donors are needed to further develop our understanding of the mechanisms used to regulate the electron transport chain in Campylobacterota. However regulation of the electron transport chain by more global cues from the environment and co-respiration are mechanism that play an important role in Campylobacterota and distinguish them from other bacteria.

## Author Contributions

All authors listed have made a substantial, direct and intellectual contribution to the work, and approved it for publication.

### Conflict of Interest Statement

The authors declare that the research was conducted in the absence of any commercial or financial relationships that could be construed as a potential conflict of interest.
